# Comparative study of attenuation of the pain caused by propofol intravenous injection, by granisetron, magnesium sulfate and nitroglycerine

**DOI:** 10.4103/1658-354X.76511

**Published:** 2011

**Authors:** Dhananjay Kumar Singh, Parul Jindal, Gaurav Singh

**Affiliations:** *Department of Anaesthesiology, Pain Management & ICU, Himalayan Institute of Medical Sciences, Jolly Grant, Dehradun, India*

**Keywords:** *Granisetron*, *magnesium sulphate*, *nitroglycerine*, *pain on injection*, *propofol*

## Abstract

**Background:**

Propofol has the disadvantage of causing pain or discomfort on injection. The aim of the study was to assess the efficacy of pretreatment with various drugs to alleviate the propofol injection pain.

**Methods:**

One hundred American Society of Anesthesiology (ASA) I and II adults, scheduled for various elective surgical procedures under general anesthesia (GA), were included in the study. They were randomly divided into four groups having 25 patients in each group. Group A received pretreatment with intravenous (i.v.) magnesium sulfate, group B received i.v. granisetron, group C received i.v. nitroglycerine and group D was the control group. One-fourth of the total calculated induction dose of propofol was administered over a period of 5 seconds. The patients were asked about the pain on injection. The intensity of pain was assessed using verbal response. A score of 0–3 which corresponds to no, mild, moderate and severe pain was recorded.

**Results:**

All the three drugs reduced the incidence and intensity of pain on propofol injection but the order of efficacy in attenuation of pain on the propofol injection was granisetron > nitroglycerine > magnesium sulfate > control.

**Conclusion:**

Granisetron was the most effective followed by nitroglycerine and magnesium sulfate in attenuating pain on propofol intravenous injection.

## INTRODUCTION

Ever since its introduction in the clinical scenario in 1977, propofol has attained unmatched popularity as an agent for intravenous (i.v.) induction. It is also used for short duration surgery, day care surgery, sedation and ambulatory surgery. But very often, it has the disadvantage of causing pain or discomfort on injection, especially when given in small veins on the dorsum of hand. This pain may be distressing to the patients and can reduce the acceptability of an otherwise useful agent. Among 33 clinical problems, propofol-induced pain ranked seventh when both clinical importance and frequency were considered.[1,2]

Incidence of pain with i.v. propofol varies between 28% and 90% in adults and 28% and 85% in children.[3] The younger the child, the higher is the incidence and intensity of pain on propofol. Many factors like site of injection, size of vein, speed of injection, buffering effect of blood, temperature of propofol and concomitant use of drugs such as local anesthetics, opiates, etc., appear to affect the incidence of pain.[4–6]

A number of both pharmacological (e.g., pre-treatment with lignocaine, ondansetron, ketorolac, nafamostat, ketamine or topical nitroglycerine application with propofol, diluting propofol with 5% dextrose or 10% intralipid and using medium- and small-chain triglycerides) and non-pharmacological methods have been used[7–14] with variable results and the research for the ideal agent to decrease pain on propofol injection is still going on.

None of the above-mentioned methods has been proved absolutely perfect for attenuation of pain due to propofol injection.

5HT_3_ antagonists like ondansetron have been found to decrease pain on propofol injection. Thus, the study was conducted with granisetron, another potent 5HT_3_ antagonist, to see its efficacy in decreasing pain on propofol injection.[9,15]

Ketamine, an N-methyl D-aspartate (NMDA) antagonist, in the subanesthetic doses, reduces the propofol injection pain by virtue of its local anesthetic property. Magnesium, another NMDA antagonist, has antinociceptive effect in humans. The effect is primarily based on regulation of calcium ion influx into the cell, the natural physiological analgesic mechanism. This is why magnesium sulfate has been taken for study to attenuate the pain by propofol injection.[16]

Nitroglycerine also has an analgesic effect on propofol injection due to its vasodilating effect when applied topically on i.v. cannulation site. The study was conducted to see the analgesic effect of i.v. nitroglycerine to alleviate pain following propofol injection.[17,18]

These proposed mechanisms prompted us to investigate the effect of pre-treatment with granisetron, magnesium sulfate and nitroglycerine on propofol injection pain.

## METHODS

After obtaining approval from hospital ethics committee and informed consent from the patients, 100 patients belonging to American Society of Anesthesiology (ASA) physical status I and II, of either sex, aged between 21 and 50 years, undergoing elective surgery under general anesthesia, were studied. Patients less than 21 years and more than 50 years, patients belonging to ASA grade III and IV, patients with history of any systemic illness, history of drug allergy to granisetron, magnesium sulfate, nitroglycerine and propofol, patients who were taking any analgesic before surgery, morbidly obese patients and patients scheduled for emergency surgery were excluded from the study.

They were randomly divided (by opening a sealed envelope) into four groups of 25 patients each. The drug solution was prepared by a co-supervisor and given to observer who dispensed 5 ml of the study drug. Group A patients received pre-treatment with i.v. magnesium sulfate (2.48 mmol diluted in 5 ml of 0.9% normal saline). Group B patients received pre-treatment with i.v. granisetron (2 mg in 5 ml of 0.9% normal saline). Group C patients received pre-treatment with i.v. nitroglycerine (200 μg in 5 ml of 0.9% normal saline). Group D patients received i.v. normal saline as a placebo (5 ml of 0.9% normal saline).

Patients of less than 21 years and more than 50 years, with ASA grade III and IV, neurological deficit, history of drug allergy to study drugs and propofol, history of taking any analgesic before surgery, history of diabetes/hypertension, history of cardiovascular/respiratory disease, morbid obesity and emergency surgery were excluded from the study. Prior to surgery, all the patients underwent thorough pre-anesthetic check-up and routine investigations like hemoglobin (Hb), total leukocyte count (TLC), differential leukocyte count (DLC), bleeding time (BT), clotting time (CT), routine urine and microscopic examination, serum creatinine, chest X-ray and electrocardiogram. If investigations were found to be within normal limits, then only the patients were selected for the study. Patients for the surgery were kept fasting for 6–8 hours and were premedicated with tab diazepam 10mg at night and 5mg 2hours prior to surgery with a sip of water.

The i.v. access was established with an 18-G cannula in a suitable vein on dorsum of non-dominant hand without any local infiltration and i.v. fluid (0.9% normal saline) was infused at 100 ml/hour. After 5 minutes, lactated Ringer’s infusion was stopped and the arm with the i.v. line was elevated for 15 seconds for gravity drainage of venous blood. Heart rate, noninvasive blood pressure, SPO_2_, end-tidal carbon dioxide and ECG were monitored. The procedure was explained to patients. No analgesic drug was given to the patient before injecting propofol. Venous occlusion was done by compressing the forearm with a tourniquet to increase the local concentration of the drug. The study drug was injected over 10 seconds and thereafter the occlusion was removed and then first 25% of the calculated dose (2.5 mg/kg) of propofol (1% w/v in lipid base) was injected over 20 seconds. Then, patients were asked to tell the observer about the severity of pain. The intensity of pain was graded using a verbal rating scale and was assessed at 0, 5, 10, 15 and 20 seconds as after 20 seconds the patient may have been under the influence of propofol.[19]

0 – None (negative response to question)

1 – Mild pain (pain reported only in response to question without any behavioral sign)

2 – Moderate pain (pain reported in response to question and accompanied by behavioral sign and pain reported spontaneously without question)

3 – Severe pain (strong vocal response or response accompanied by facial grimacing, arm withdrawal and tears)

The observer was blinded to the drug being given to the patient. Thereafter, induction of anesthesia was continued with the rest of the calculated propofol dose, and for analgesia, fentanyl 2 μg/kg was given to all patients. The patient was intubated with appropriate size endotracheal tube after giving vecuronium. Anesthesia was maintained with isoflurane and nitrous oxide–oxygen (66–33%).

Data collected were subjected to standard statistical analysis. Descriptive statistics such as range, mean, standard deviation (SD) were used to summarize the baseline clinical and demographic profile of the patients. Categorical data were analyzed using chi-square (χ^2^) test.

## RESULTS

There was no significant difference in demographic profile of the patients in all the groups [Table 1]. Majority of patients were males (59%) in all the groups. Majority of patients in all the groups (81%) belonged to ASA Grade I.

**Table 1:** Demographic profile

	Group A	Group B	Group C	Group D
Age in years	33.44 ±	34.54 ±	36.08 ±	33.08 ±
Mean ± SD	7.52	9.03	9.24	10.02
Male:female	14:11	13:12	15:10	16:9
ASA Grade I:II	20:5	21:4	19:6	21:4

**Figure 1:**
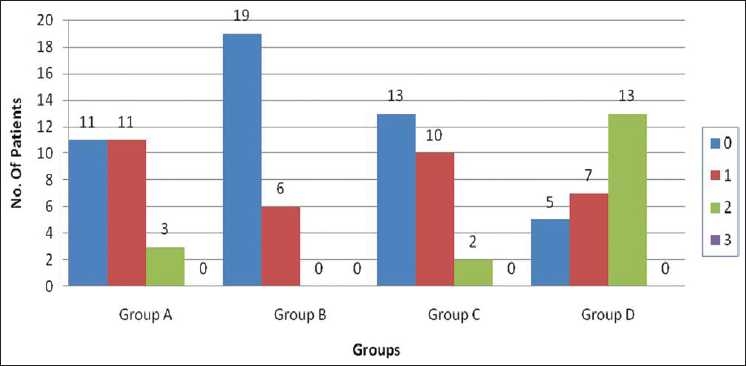
Pain score of patients at 5 seconds

The number of patients with grade 0 pain at 5 seconds was 11, 19, 13 and 5 in groups A, B, C and D, respectively. In our study, pain at 5 seconds showed statistically significant difference in pain relief in group A, group B and group C, when compared with group D. Statistical analysis (*P* < 0.005) among all the three groups – groups A, B and C was highly significant [Figure 1].

**Figure 2:**
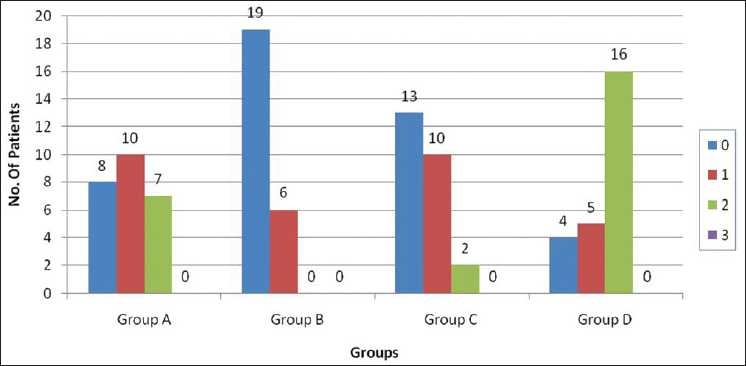
Pain score of patients at 10 seconds

The number of patients with grade 0 pain at 10 seconds was 8, 19, 13 and 4 in groups A, B, C and D, respectively. At 10 seconds, there was no statistical significant difference when groups A and C were compared. Statistical analysis among all the three groups – groups A, B and C was significant (*P* < 0.05) [Figure 2].

**Figure 3:**
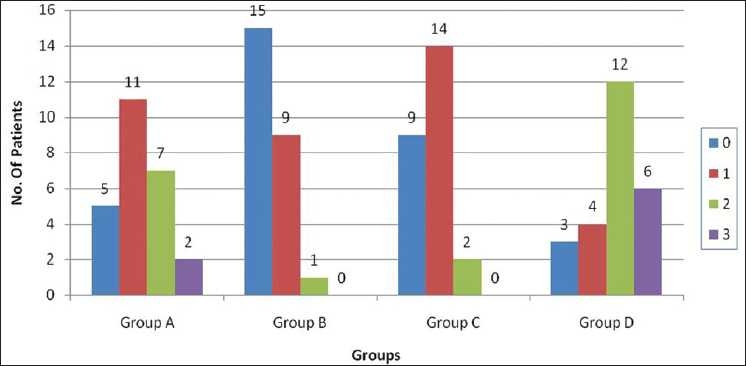
Pain score of patients at 15 seconds

**Figure 4:**
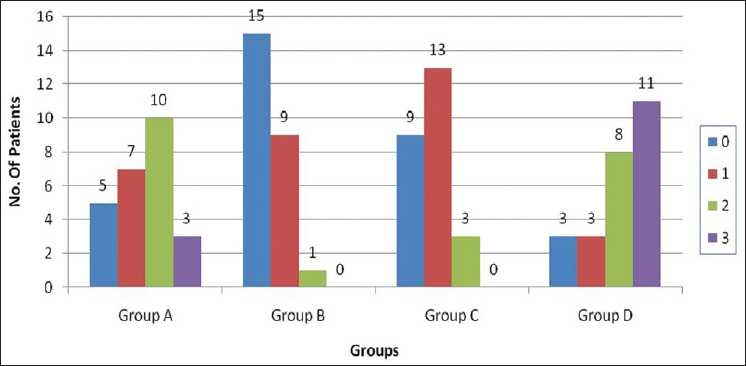
Pain score of patients at 20 seconds

The number of patients with grade 0 pain at 15 seconds was 5, 15, 9 and 3 in groups A, B, C and D, respectively. There were no significant statistical differences when groups A, B and C were compared among themselves [Figure 3]. The number of patients with grade 0 pain at 20 seconds was 5, 15, 9 and 3 in groups A, B, C and D, respectively. When group A was compared with groups B and C, there was significant difference but on comparing group B and group C (*P* > 0.05), there was no significant statistical difference. Statistical analysis (*P* < 0.05) among all the three groups – groups A, B and C was highly significant [Figure 4]. The order of efficacy of drugs at different time intervals is summarized in [Table 2].

**Table 2:** Efficacy of drugs at different time intervals

Time interval (seconds)	Order of efficacy
5	Granisetron (24%) > NTG (48%) > MgSO4 (56%)
10	Granisetron (24%) > NTG (48%) > MgSO4 (68%)
15	Granisetron (40%) > NTG (64%) > MgSO4 (80%)
20	Granisetron (40%) > NTG (64%) > MgSO4 (80%)

NTG, nitroglycerin

In group A, 12% patients experienced nausea and shivering each. In group B, there were no significant postoperative complications, except for shivering in 4% patients. In group C, 8% patients experienced nausea, 4% had vomiting and hypothermia and 16% had shivering. These were much less in comparison to group D (control group). In group D, 8% patients experienced nausea, 12% patients had vomiting and 24% patients had hypothermia and shivering.

## DISCUSSION

The incidence of pain caused by propofol injection was reported to range from 28 to 90% in adults. Propofol belongs to the group of phenols that can irritate the skin, mucous membrane, and venous intima.[20–21] The possible explanation for the pain includes endothelial irritation, osmolality differences, unphysiological pH and the activation of pain mediators.

Scott *et al*. speculated that the injection pain is caused by activation of the Kallikrein-Kinin system either by propofol or by the lipid solvent, thereby generating kinins, probably bradykinin.[6] Bradykinin, by causing local vasodilation and hyperpermeability, may increase the contact between the aqueous phase propofol and the free nerve ending, resulting in pain on injection. This pain has a 10–20 seconds delayed onset.[22]

A 4-point verbal categorical scoring system was chosen in this study rather than visual analogue score (VAS) as it was very simple to be used by the patient and also as appropriate hand eye coordination required for a VAS might not be present in all patients during the rapidly changing state of consciousness during induction.[19]

Granisetron reduced the incidence of propofol injection pain to 24% at 5 seconds, 24% at 10 seconds, 40% at 15 seconds and 40% at 20 seconds in comparison to the control group, whereas placebo had an incidence of 80% at 5 seconds, 84% at 10 seconds, 88% at 15 seconds and 88% at 20 seconds and showed statistical significance. Ambesh *et al*. reported that the incidence of pain was decreased to 25% in the ondansetron group in comparison to 55% in the saline group.[9] Ye *et al*. suggested that this was due to dual mechanism of action of ondansetron as a sodium-channel blocker and 5HT_3_ receptor antagonist (peripheral 5HT_3_ receptors involve nociceptive pathways). The exact mechanism of alleviation of propofol injection pain by granisetron is not known. This may have been the result of a peripheral local anesthetic action, which attenuated the afferent pain pathway rather than a central analgesic effect, similar to the mechanism of ondansetron (i.e., blockage of sodium channel and antagonism of 5HT_3_ receptor).[23]

In our study, magnesium sulfate significantly reduced the incidence of pain up to 56% at 5 seconds, 68% at 10 seconds and 80% at 15 and 20 seconds, whereas the control group had an incidence of 80% at 5 seconds, 84% at 10 seconds, 88% at 15 seconds and 88% at 20 seconds and showed statistical significance. Dilek *et al*. concluded that pre-treatment with magnesium sulfate reduces the incidence to 36% in comparison to control saline group which has incidence of 86% on pain on propofol i.v. injection.[16] Calcium is important for the release of neurotransmitters and other substances implicated in nociception and inflammation. Magnesium acts as a non-competitive inhibitor of the inositol 1,[4] 5-triphosphate (IP_3_)-gated calcium channel and of IP_3_ binding. Therefore, it may be considered as an intracellular calcium antagonist. It also has a role as a calcium antagonist at the ryanodine subgroup of calcium release channel receptors in the sarcoplasmic reticulum.[24] The second possible explanation for the analgesic action of magnesium is to block the NMDA receptor which is coupled to an ion channel permeable to K^+^ and Ca ^+^ in a voltage-dependent manner.[25] Magnesium also has a vasodilatory effect mediated by endothelium-derived nitric oxide.[13] This mechanism might also explain the ability of magnesium to reduce pain on the injection of propofol.

In this study, pre-treatment with i.v. nitroglycerine reduced the incidence up to 48% at 5 seconds, 48% in 10 seconds, 64% in 15 seconds and 64% in 20 seconds in comparison to control group which had incidence of 80% at 5 seconds, 84% in 10 seconds, 88% in 15 seconds and 88% in 20 seconds and showed statistical significance. Wilkinson *et al*. reported that 18 patients (67%) pretreated with nitroglycerine experienced no pain compared with 10 (33%) in the placebo group in a study conducted in 60 patients.[17] The vein size might be an important factor in determining the pain during propofol injection. Injecting propofol in large antecubital vein caused no pain,[7] injecting it into midsize forearm vein caused only a 2.5% incidence of pain,[13] while injecting it into the small vein on the dorsum of the hand led to a 37.5% incidence of pain.[13] Therefore, vein size, vasospasm, and perhaps blood flow might be important in determining painful injections. Nitroglycerine reduces the pain by decreasing the contact of drug within the vessel wall by venodilatation.[7] Lohmann *et al*. demonstrated dilation of the vein by more than 50% in over half the subjects treated with nitroglycerin within 15 minutes of application.[26]

The present study postulates that pain on propofol i.v. injection can be attenuated by use of different drugs. Among them, granisetron was found to be the most effective than nitroglycerine followed by magnesium sulphate, without any significant postoperative complications. Maximum number of patients who had no pain belonged to group B, i.e., granisetron group and severe pain score was recorded in maximum numbers of patients in group D, i.e., placebo group, followed by group A, i.e., magnesium sulfate group.

## References

[1] Smith I, White PF, Nathansun M, Gouldson R (1994). Propofol: An update on its clinical use. Anaesthesiology.

[2] Macario A, Wringer M, Truong P, Lee M (1999). Which clinical anaesthesia outcomes are both common and important to avoid. The perspective of a panel of expert anaesthesiologists?. Anaesth Analg.

[3] Stark RD, Binks S, Dutka VN, Arnstein MJ, Glen JB (1955). A review of the safety and tolerance of propofol. Postgrad Med J.

[4] Mangar D, Holak EJ (1992). Tourniquet at 50 mmHg followed by intravenous lidocaine diminishes hand pain associated with propofol injection. Anaesth Analg.

[5] Valtonen M, Lisalo E, Kanto J, Rosenberg P (1989). Propofol as an induction agent in children: Pain on injection and pharmacokinetics. Acta Anaesthesiol Scand.

[6] Scott RP, Sunders DA, Norman J (1988). Propofol: Clinical strategies for preventing the pain on injection. Anaesthesia.

[7] Mattila MA, Koski EM (1985). Venous sequelae after intravenous propofol: A comparison with methohexitone in short anaesthesia. Postgrad Med J.

[8] King SY, Davis FM, Wells JE (1992). Lidocaine for the prevention of pain due to injection of propofol. Anesth Analg.

[9] Ambesh SP, Dubey PK, Sinha PK (1999). Ondansetron pretreatment to alleviate pain on propofol injection: A randomized, controlled, double blind study. Anaesth Analg.

[10] Johnson RA, Harper NJ, Chadwick S, Vohra A (1990). Pain of injection of propofol: Methods of alleviation. Anaesthesia.

[11] McCulloh MJ, Lees NW (1985). Assessment and modification of pain on induction with propofol. Anaesthesia.

[12] McCrirrick A, Hunter S (1990). Pain on injection of propofol: The effect of injectate temperature. Anaesthesia.

[13] Barker P, Langton JA, Murphy P, Rowbotham DJ (1991). Effect of prior administration of cold saline on pain during propofol injection: A comparison with cold propofol and propofol with lignocaine. Anaesthesia.

[14] Klement W, Arndt JO (1991). Pain on injection of propofol: Effects of concentration and diluent. Br J Anaesth.

[15] Dubey PK, Sureshwar P (2003). Pain on injection of propofol: The effect of granisetron pretreatment. Clin J Pain.

[16] Dilek M, Turan A, Karamamanlioglu B, Necdet S, Pamukcu Z (2002). The use of magnesium sulfate to prevent pain on injection of propofol. Anaesth Analg.

[17] Wilkinson D, Anderson M, Gauntlett IS (1993). Pain on injection of propofol: Modification by Nitroglycerin. Anesth Analg.

[18] Selda SB, Osman N, Mustafa O (2006). Analgesic effect of nitroglycerine added to lidocaine on intravenous regional anaesthesia. Anaesth Analg.

[19] Ohnhaus EE, Adler R (1975). Methodological problem in the measurement of pain: A comparison between the verbal rating scale and the visual analogue scale. Pain.

[20] Hynynen M, Korttila K, Tammisto T (1985). Pain on IV injection of propofol (ICI 35868) in emulsion formulation: Short communication. Acta Anaesthesiol Scand.

[21] Akeson J (2008). Pain on injection of propofol - Why bother.[Editorial]?. Acta Anaesthesiol Scand.

[22] Coderre TJ, Katz J, Vaccarino AL, Melcack R (1993). Contribution of central neuroplasticity to pathological pain: Review of clinical and experimental evidence. Pain.

[23] Ye JH, Mui WC, Ren J (1997). Ondansetron exhibit the properties of a local anesthetic. Anaesth Analg.

[24] Altura BT, Altura BM (1987). Endothelium dependent relaxation in coronary arteries requires magnesium ions. Br J Pharmacol.

[25] Katzung BG, Chatterjee K, Katzung BG (1998). Vasodilators and the treatment of angina pectoris. Basic and clinical pharmacology.

[26] Lohmann M, Moller I, Brynitz S, Bjerrum OW (1984). Nitroglycerin ointment as aid to venepuncture. Lancet.

